# Impact of Buriti Oil from *Mauritia flexuosa* Palm Tree on the Rheological, Thermal, and Mechanical Properties of Linear Low-Density Polyethylene for Improved Sustainability

**DOI:** 10.3390/polym16213037

**Published:** 2024-10-29

**Authors:** Odilon Leite-Barbosa, Marcelo Ferreira Leão de Oliveira, Fernanda Cristina Fernandes Braga, Sergio Neves Monteiro, Marcia Gomes de Oliveira, Valdir Florêncio Veiga-Junior

**Affiliations:** 1 Materials Science Department, Military Institute of Engineering, Praça General Tibúrcio, Rio de Janeiro 22290-270, RJ, Brazil; odilonleitebc@gmail.com (O.L.-B.); snevesmonteiro@gmail.com (S.N.M.); 2 Division of Materials, National Institute of Technology—INT, Avenida Venezuela 82, Saúde, Rio de Janeiro 20081-312, RJ, Brazil; marceloilha@gmail.com (M.F.L.d.O.); fernanda.braga@int.gov.br (F.C.F.B.)

**Keywords:** buriti oil, plasticizer, LLDPE (linear low-density polyethylene), rheological properties, thermal properties, mechanical properties

## Abstract

Recent advancements highlight the utilization of vegetable oils as additives in polymeric materials, particularly for replacing conventional plasticizers. Buriti oil (BO), extracted from the Amazon’s *Mauritia flexuosa* palm tree fruit, boasts an impressive profile of vitamins, minerals, proteins, carotenoids, and tocopherol. This study investigates the impact of incorporating buriti oil as a plasticizer in linear low-density polyethylene (LLDPE) matrices. The aim of this research was to evaluate how buriti oil, a bioactive compound, influences the thermal and rheological properties of LLDPE. Buriti oil/LLDPE compositions were prepared via melt intercalation techniques, and the resulting materials were characterized through thermogravimetric analysis (TGA), differential scanning calorimetry (DSC), Fourier-transform infrared (FTIR) spectroscopy, scanning electron microscopy (SEM), mechanical property testing, and contact angle measurement. The addition of buriti oil was found to act as a processing aid and plasticizer, enhancing the fluidity of LLDPE polymer chains. TGA revealed distinct thermal stabilities for buriti oil/LLDPE under different degradation conditions. Notably, buriti oil exhibited an initial weight loss temperature of 402 °C, whereas that of LLDPE was 466.4 °C. This indicated a minor reduction in the thermal stability of buriti oil/LLDPE compositions. The thermal stability, as observed through DSC, displayed a nuanced response to the oil’s incorporation, suggesting a complex interaction between the oil and polymer matrix. Detailed mechanical testing indicated a marked increase in tensile strength and elongation at break, especially at optimal concentrations of buriti oil. SEM analysis showcased a more uniform and less brittle microstructure, correlating with the enhanced mechanical properties. Contact angle measurements revealed a notable shift in surface hydrophobicity, indicating a change in the surface chemistry. This study demonstrates that buriti oil can positively influence the processability and thermal properties of LLDPE, thus expanding its potential applications as an effective plasticizer.

## 1. Introduction

The environmental problems associated with conventional plasticizers have sparked a fervent search for sustainable alternatives in the field of polymer science. As the need for biodegradable materials becomes increasingly urgent, the exploration of biomass-based additives stands out as a promising approach to mitigate the environmental impact of polymeric materials [1,2]. Among these additives, the integration of natural oils as plasticizers has gained considerable prominence in recent years due to several advantages [3].

The International Union of Pure and Applied Chemistry (IUPAC) defines plasticizers as substances added to polymers to enhance certain properties, such as flexibility, malleability, and other characteristics [4]. These additives play a crucial role in modifying the performance characteristics of polymers, allowing their use in a wide range of applications where these properties are essential [5]. Most plasticizers are composed of colorless, odorless liquids, often esters, which are used in mixtures in various proportions. Although their primary function is to improve the processability and mechanical properties of polymers, their presence is so significant that they are considered an integral part of formulations rather than mere additives [5,6].

Historically, thousands of substances have been tested as plasticizers, but fewer than 100 have commercial importance due to performance, cost, and regulatory compliance. The global plasticizer market is closely linked to the polymer market, particularly polyvinyl chloride (PVC), where more than 90% of plasticizers are used [7,8]. However, concerns over the environmental impact of synthetic plasticizers, such as phthalates, have grown due to their fossil-based origin and low biodegradability [9]. These plasticizers can persist in the environment, accumulating and releasing toxic substances, especially under heat or mechanical stress. Additionally, the migration of these compounds out of polymers degrades both the material’s performance and environmental safety. Stricter regulations are limiting their use due to health and environmental risks, highlighting the urgent need for sustainable alternatives [2,10].

As regulations and consumer demand for safer and more sustainable alternatives continue to grow, biomass-based plasticizers, such as buriti, palm, and copaiba oils, are gaining increasing importance. These natural additives offer a viable way to enhance the environmental sustainability of polymers while preserving or improving their mechanical performance [11,12]. In being derived from renewable sources, they help reduce dependence on fossil fuels, contributing to a more sustainable production cycle. Additionally, their biodegradability ensures they decompose more easily in the environment, reducing long-term pollution. These characteristics make biomass-derived plasticizers ideal for lowering the ecological footprint of polymer products, all while providing the necessary flexibility and durability. Furthermore, natural oils tend to be less toxic than synthetic alternatives, aligning with regulatory standards and meeting the growing demand for eco-friendly materials [13,14,15].

Among the myriads of natural oils, buriti oil, extracted from the fruit of the traditional Amazonian *Mauritia flexuosa* palm tree, stands out for its rich content of vitamins, minerals, proteins, carotenoids, and tocopherol. The multifaceted benefits attributed to buriti oil, ranging from antioxidant activity to high oxidative stability, position it as a compelling bio-additive for polymeric materials [16]. Buriti oil can be considered a primary plasticizer, as it can induce softness and elongation when added to the polymer, without the need for other components to enhance its effectiveness [7,17]. Additionally, due to its bioactive properties, buriti oil may offer additional advantages, such as increased thermal stability and oxidation resistance, essential characteristics for applications requiring durability and performance under adverse environmental conditions [13,14,15].

The prevailing trend indicates a shift towards ecological plasticizers potentially replacing synthetic counterparts entirely [18]. Flexibility, workability, and distensibility are crucial properties for polymers as they directly influence their adaptability to various processing conditions and end-use applications. Flexibility enables the polymer to conform to diverse shapes, while workability facilitates ease of handling during production, and distensibility is essential for withstanding deformations without compromising structural integrity. These characteristics are fundamental for a polymer’s applicability and performance across a wide range of industrial applications [19,20]. Another critical aspect in the selection of a plasticizer is its compatibility with the polymer matrix. Inadequate compatibility can lead to plasticizer extraction, compromising the performance of the final material and, in some cases, endangering the health of end-users [21].

The demand for materials that reduce reliance on conventional additives is also relevant for widely used polymers like linear low-density polyethylene (LLDPE). LLDPE, a thermoplastic commonly employed in flexible packaging, films, and pipes, is prized for its flexibility, transparency, and ease of processing. These attributes make it a preferred material in a variety of applications, from packaging to industrial uses [22]. However, de-spite its advantages, LLDPE faces certain limitations, such as its relatively low thermal stability and moderate mechanical strength, which need to be addressed to meet the durability and sustainability requirements of industrial applications [23,24]. There is a growing need to enhance the processability, mechanical performance, and environmental impact of LLDPE, driving research into innovative solutions that can overcome these inherent challenges [25,26].

Numerous studies are currently underway to develop ecological plasticizers, and many oils stand out when used as plasticizers, notable for their biodegradability, low toxicity, and renewability. For instance, palm oil has been successfully used as a plasticizer in natural rubber (NR) and carbon black (CB) composites, resulting in enhanced properties such as improved CB dispersion and increased tensile strength, especially at lower oil contents compared to traditional petroleum-based plasticizers [27]. Similarly, soybean oil derivatives, particularly methoxy polyethylene glycol-modified epoxidized soybean oil (mPEG-ESBO), have shown promise in PVC applications, providing superior tensile strength and elongation while lowering the glass transition temperature (Tg) compared to conventional phthalate plasticizers [28]. Additionally, Moringa oleifera oil has been investigated as a bioplasticizer for natural rubber vulcanizates, where it has demonstrated improved cure rate, better mechanical properties, and enhanced rolling resistance and wet grip behavior, positioning it as a viable green alternative to naphthenic oil in rubber formulations [22].

This investigation aims to comprehensively evaluate the influence of buriti oil, extracted from the fruits of the *Mauritia flexuosa* palm tree, on the rheological and thermal properties of LLDPE, crucial aspects governing the material’s processability and performance. With a particular emphasis on sustainable alternatives, the study seeks to elucidate the role of buriti oil in improving the fluidity of LLDPE polymer chains, subsequently impacting thermal stability and oxidative resistance. The unique attributes of buriti oil, coupled with its versatile applications ranging from cosmetics to food, underscore the potential significance of this research in advancing sustainable practices within the realm of polymeric materials. Through a systematic analysis of processing parameters, thermal behavior, and rheological characteristics, this study aspires to disclose valuable insights to the growing body of knowledge surrounding bio-additives in polymer science.

## 2. Materials and Methods

### 2.1. Materials

In this study, linear low-density polyethylene (LLDPE), trade name Dowlex™ GM8480G, was used as the polymer matrix. The LLDPE, acquired from Dow Chemical Company, has a density of 0.917 g/cm^3^ and a melt flow index (MFI) of 3.0 g/10 min (measured at 230 °C with a 2.16 kg load), in accordance with the ASTM D-1238 standard [29]. Buriti oil, produced in September 2019 and provided by Rio Preto Agroindustrial da Amazônia Ltd.a, from Rio Preto da Eva, Amazonas State, Brazil, was used as an additive. Both materials were prepared before processing. The LLDPE granules were dried in an oven at 70 °C for 24 h to eliminate any moisture, ensuring optimal mixing and preventing interference in the interfacial adhesion of the composites. The oil was used as received without further purification.

This work was registered in the National Management System of Genetic Heritage and Associated Traditional Knowledge (SISGEN) under number A5F27C0.

### 2.2. Methods

#### 2.2.1. Processing of LLDPE and LLDPE/Buriti Oil

LLDPE compositions, incorporating various percentages of buriti oil, were processed using melt intercalation. The compositions were blended at 150 °C and 80 rpm for 7 min in a Haake Polylab torque rheometer connected to a Rheomix mixing chamber equipped with roller rotors (70% fill factor). The addition of the filler occurred after 2 min in a batch mixer. Table 1 shows a detailed overview of the compositions studied.

#### 2.2.2. Preparation of LLDPE and LLDPE/Buriti Oil Specimens

The preparation of the LLDPE and LLDPE/buriti oil specimens involved several key steps to ensure uniformity and consistency in the final samples. Initially, the compositions were ground using a SEIBT two-knife mill (model MGHS 1.5/85) operating at a rotation speed of 1150 rpm. This milling process was essential to reduce the particle size of the material, ensuring homogeneous mixing of the LLDPE and buriti oil. Following the milling step, the ground compositions were subjected to molding using a heated hydraulic press. The molding process was conducted at a temperature of 150 °C for a duration of 10 min, applying a pressure of 10 t. This temperature was selected based on the melting point of LLDPE, ensuring proper fusion and integration of the buriti oil within the polymer matrix. Once the molding step was completed, the samples were cooled using a water-circulated press to bring the temperature down to 30 °C while maintaining the applied pressure of 10 tons. This rapid cooling process helped to stabilize the polymer structure and prevent any deformation or warping in the final product. The final result was polymeric plates with dimensions of 100 × 100 mm and a thickness of 3 mm. These plates were subsequently used for cutting test specimens according to the required dimensions for mechanical, thermal, and other characterization tests.

#### 2.2.3. Fourier-Transform Infrared (FTIR) Spectroscopy

The chemical structure and bonding interactions in the LLDPE and LLDPE/buriti oil compositions were analyzed by FTIR spectroscopy using a Nicolet Nexus 470 spectrometer in transmission mode. The samples were scanned in the range of 4000–400 cm^−1^ at a spectral resolution of 4 cm^−1^ using 64 scans. The buriti oil was mixed with dried potassium bromide (KBr) powder and compressed into a disc.

#### 2.2.4. Thermogravimetric Analysis (TGA)

TGA and its derivative curves (TG/DTG) of the weight loss versus temperature of buriti oil, LLDPE, and LLDPE compositions was performed on a STA 409 PC Luxx thermogravimetric analyzer from NETZSCH, using approximately 10 mg of sample weighed in an aluminum pan, operating at temperatures ranging from 30 °C to 560 °C at a heating rate of 20 °C/min under a nitrogen atmosphere with a steady flow of 60 mL/min. 

#### 2.2.5. Differential Scanning Calorimetry (DSC)

Thermal analyses were also performed using a DSC Q100 calorimeter (TA Instruments). Nitrogen was used as purge gas at a flow rate of 20 mL.min^−1^. Samples of about 5 mg were weighed and used in the analysis. They were first heated from 20 °C to 200 °C at a rate of 10 °C.min^−1^ to eliminate the thermal history and subsequently cooled to 20 °C at a rate of 10 °C.min^−1^. The second heating cycle was conducted using the same conditions as the first cycle. From the second heating cycle’s curve, it was possible to obtain the cold crystallization temperature (T_cc_), the melting temperature (T_m_), the melting enthalpy (ΔH_f_), and the cold crystallization enthalpy (ΔH_cc_).

The degree of crystallinity was determined by Equation (1):

(1) $$X_{c}(\%)=\frac{∆H_{m}}{w\times ∆{H_{m}}^{0}}\times 100$$

where ΔH_m_ is obtained from the area of the endothermic peak, ΔH_m_^0^ is the enthalpy of fusion of the pure substance with 100% crystallinity, and w is the mass fraction of oil in the compositions. The value of ΔH_m_^0^ for linear low-density polyethylene (LLDPE) is 293 J/g [30].

#### 2.2.6. Scanning Electron Microscopy (SEM)

The morphology of the processed compositions’ surfaces was observed via a FEI scanning electron microscope, model Inspect S 50, in low vacuum mode using backscattered electron detectors (BEI), at an acceleration voltage of 15 kV. The samples were fixed on aluminum supports and coated with platinum in an EMITEC metalizing apparatus, model SC 7620, at 20 mA for 2 min. The SEM images were collected at a magnification of 1000×.

#### 2.2.7. Mechanical Properties

Tensile tests of the LLDPE and LLDPE compositions were carried out according to DIN 53504 specifications on an EMIC universal tester, model DL 3000, at 23 °C and with a crosshead speed of 100 mm/min. Dumbbell-shaped specimens were prepared by compression molding at a temperature of 150 °C and with 10-ton force for 10 min, using a MARCONI hydraulic press, model MA098. Five samples were tested from each composition and the average values were reported.

#### 2.2.8. Hydrophobicity (Contact Angle Measurement)

The surface hydrophobicity of LLDPE and compositions was characterized by contact angle and conducted in a Pocket Goniometer (model PGX+). The water was utilized with proper solvent for analyses of samples.

## 3. Results and Discussion

### 3.1. Fourier-Transform Infrared (FTIR) Spectroscopy

**Figure 1:**
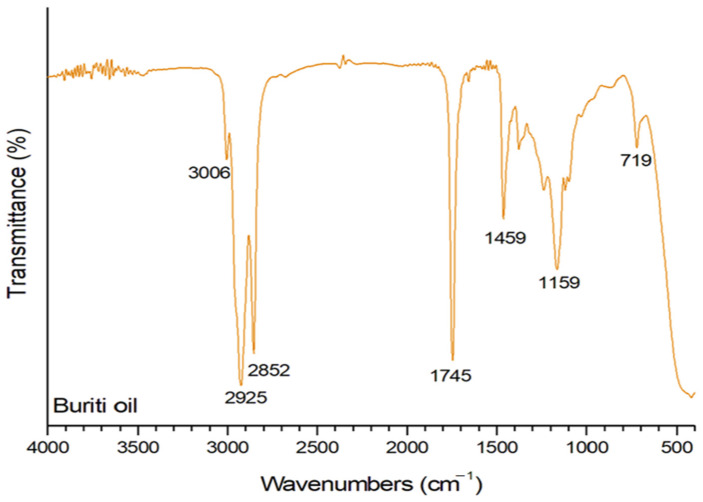
FTIR spectra of buriti oil.

The FTIR spectrum of buriti oil, shown in Figure 1, reveals distinct absorption bands, as observed in previous studies on similar materials. The spectrum exhibits significant bands associated with saturated alkanes, such as the C–H stretching at 2925 cm^−1^ and 2852 cm^−1^. The band at 1745 cm^−1^ denotes the C=O stretching vibration of carboxylic groups, suggesting the occurrence of carbonyl groups in fatty acid esters [31]. In the range of 1100–1500 cm^−1^, the absorption profile is influenced by several bands, with contributions from oleic acid (OA) and some from angular triolein (AT). The bands at 2852 cm^−1^ and 1159 cm^−1^ can be attributed to aliphatic hydrocarbons and C–H bending vibrations, respectively. These bands contribute to the characterization of long hydrocarbon chains and the scissoring stresses in the C–H2 groups, aligning with the structural characteristics of fatty acids in buriti oil [32,33].

The bands at 1459 cm^−1^ and 719 cm^−1^ correspond to C–H2 scissoring and asymmetric C–H3 stretching, respectively. These bands further elucidate the molecular structure of buriti oil, providing insights into the specific vibrations associated with its aliphatic hydrocarbons [34,35]. The band at 3006 cm^−1^ is attributed to C–H stretching related to =C–H bonding. The observed spectral features align with the expected characteristics of long-chain fatty acids found in buriti oil. The presence of ester functions, unsaturation, and specific vibrational modes corroborates the typical composition of vegetable oils. The spectral similarities with oleic and palmitic acids further validate the fatty acid composition of buriti oil. Detailed analyses reveal specific vibrations related to the molecular structure of buriti oil. Bands associated with C=C–C–O stretching and C–C movement provide insights into the molecular arrangement and potential similarities with triglycerides such as triolein [34,36].

The FTIR spectrum of buriti oil shows characteristic bands, such as the C–H stretches at 2925 cm^−1^ and 2852 cm^−1^ and the C=O vibration at 1745 cm^−1^, which indicate the presence of both saturated and unsaturated fatty acids, such as oleic and palmitic acids. These observations are consistent with the findings of Albuquerque et al. [31], who also identified these bands in buriti oil due to the presence of long-chain fatty acids and triglycerides. Buriti oil is a multicomponent mixture predominantly composed of triglycerides, as reported by Silva et al. [37], who found that it mainly consists of triacylglycerols (91.4%) and relevant levels of mono- and diacylglycerols. The fatty acid composition, particularly regarding oleic and palmitic acids, is similar to that of olive oil and contributes to the plasticity of the material, playing a role similar to other vegetable oils, such as soybean and castor oils, which are widely used as bio-based plasticizers in polymers due to their ability to enhance flexibility [38,39].

The presence of these fatty acids is crucial for the plasticizing function of buriti oil in LLDPE. The main components, such as fatty acids, act as physical plasticizers, reducing the stiffness of polymer chains, as evidenced by the decrease in viscosity and torque during processing. These acids promote greater fluidity in the material due to their ability to reduce intermolecular interactions in the polyethylene chains without chemically modifying the polymer matrix [40,41].

Like buriti oil, other vegetable oils, such as castor oil [42] and tung oil [43], also exhibit plasticizing behavior in polymers. Khalaf et al. [38] demonstrated that the addition of vegetable oils to nitrile butadiene rubber (NBR) significantly improves processability and mechanical properties due to the presence of saturated and unsaturated fatty acids. The similar chemical composition of these oils, with the presence of functional groups such as esters and double bonds, is also found in buriti oil. These groups act as internal lubricating agents, which may explain the reduced torque observed during LLDPE processing [38,44]. Similarly, studies on epoxidized oils, such as soybean oil, show that the presence of double bonds and epoxy groups enhances plasticization by reducing intermolecular forces in the polymer [44]. This property is also attributed to buriti oil, especially due to the presence of unsaturated fatty acids, such as oleic acid.

In addition to its predominant composition of fatty acids and triglycerides, it is important to highlight that to enhance the efficiency of buriti oil as a plasticizer, it can undergo chemical modifications, such as epoxidation. Epoxidation introduces epoxy groups into the double bonds of the fatty acids, increasing compatibility with polymers such as LLDPE and improving thermal stability and resistance to migration. This type of modification has been widely applied to other vegetable oils and has shown significant results in improving plasticizing capabilities in polymers, making the oil more suitable for industrial applications [40,44].

### 3.2. Torque-Specific Energy–Time Analysis

**Figure 2:**
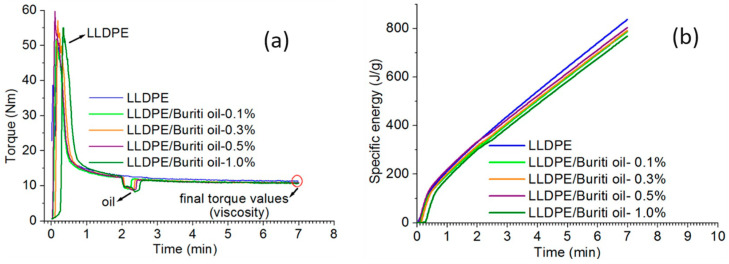
Torque (**a**) and specific energy (**b**) curves as a function of processing time for the LLDPE and compositions.

The torque and specific energy values, along with the corresponding curves depicted in Figure 2 and Table 2, offer insights into the processing behavior of LLDPE and its compositions with buriti oil (BO).

The torque curves (Figure 2a) demonstrate how torque varies with processing time. Notably, the addition of buriti oil led to a slight reduction in the final torque values. This reduction indicates a subtle decrease in viscosity, emphasizing the impact of oil incorporation on material flow. The torque–time curves for LLDPE exhibited a single loading peak, suggesting a uniform loading process. However, upon introducing buriti oil, a viscosity reduction became apparent, noticeable in the torque decrease after the loading peak. The recorded torque peak at the beginning of mixing is attributed to friction generated between solids (particle–particle and particle–wall) and the plastic deformation of polymeric particles. Subsequently, a torque decrease occurs, linked to polymer material fusion. The reduction in viscosity post-fusion renders the material more fluid, leading to a decrease in force required for rotor movement. The torque stabilizes towards the end of processing, indicating consistent viscosity and suggesting improved processability with the addition of buriti oil [45].

Although the addition of 1% buriti oil has shown favorable results, higher concentrations, such as 2% or 5%, could lead to the saturation of the polymer matrix. This may result in reduced cohesion between polymer chains, lowering mechanical strength and increasing the migration of the plasticizer to the surface of the material, thereby compromising its thermal and mechanical properties [10]. Future studies are necessary to determine the optimal concentration of buriti oil without negatively affecting the polymer’s integrity.

Thus, the addition of buriti oil (BO) demonstrated a 6.1% reduction in torque when using 1% BO in LLDPE, indicating improved processability with a relatively small amount of plasticizer. Additionally, a reduction of 8.3% in specific energy was observed, from 838 J/g to 768.4 J/g, which also reflects a significant improvement in processability. When compared to other studies, it is evident that the use of higher percentages of plasticizers also resulted in significant torque reductions. For example, in Khalaf et al. [38], conventional plasticizers like dioctyl phthalate (DOP) and vegetable oils, such as olive and orange oils, were used at concentrations of 5% (5 phr), leading to more-pronounced reductions in the torque of their NBR mixtures. Furthermore, in the study by Rapa et al. [46] with PLA and plasticizers such as DBEEA and TBC, both bioplastics, the reduction in torque occurred subtly, with blends containing up to 10% and 20% plasticizer. The results were similar to those of buriti oil, although the plasticizer concentrations were considerably higher. This highlights the effectiveness of buriti oil at lower concentrations, whereas other studies required higher concentrations to achieve similar effects on processability.

The specific energy curves (Figure 2b) showcase the cumulative energy demand for mixing over time. Initially, there is a substantial increase in energy demand within a short timeframe, reflecting the need to mix the polymer in its solid state. Following polymer fusion, the energy demand shows a nearly constant growth. Importantly, compositions with buriti oil exhibit lower torque and energy demand values, indicating enhanced mechanical workability and a potential plasticizing effect. Both torque and specific energy analyses support the notion that buriti oil improves the processability of LLDPE. The reduced torque values and energy demand imply increased fluidity, suggesting a plastifying effect of the buriti oil. This improvement in processability is consistent across all compositions, with the 1%-buriti-oil composition demonstrating the most favorable results. These findings align with the expected behavior of a plastifying agent, emphasizing the potential of buriti oil as an enhancer in the processing of LLDPE [47].

### 3.3. Thermogravimetry Analysis (TGA)

**Figure 3:**
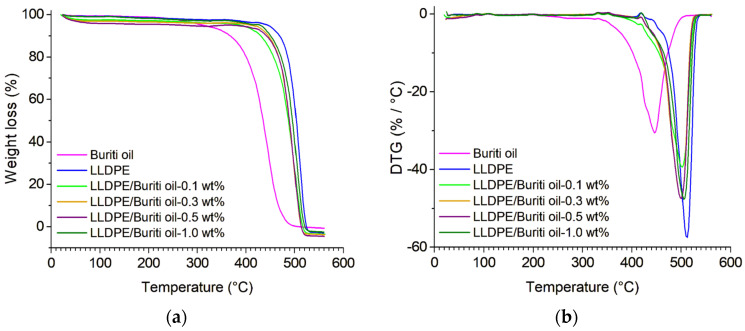
Thermogravimetric analysis (**a**) and differential thermal gravimetry (**b**) of buriti oil, pure LLDPE, and LLDPE/buriti oil compositions.

The thermogravimetric decomposition for the compositions, expressed in terms of weight loss as a function of temperature, is shown in Figure 3. It is very interesting to note that the thermal stabilities of buriti oil and LLDPE under different degradation test conditions have shown distinct initial temperatures upon weight loss (Tonset).

The TGA curves revealed that all samples exhibited gradual weight loss, with a nearly imperceptible onset of degradation for each sample. However, there was variation in the Tonset among the samples, indicating different thermal behaviors. A slight reduction in Tonset was noted for the LLDPE/buriti oil composition with 0.1 wt%, and a slight increase in Tonset values was observed for other polyethylene compositions with increasing levels of buriti oil when subjected to the same analysis conditions. Buriti oil exhibited a distinctive band in the range of 370 °C to 470 °C, indicating specific decomposition in this temperature range [36,45]. In the LLDPE/buriti samples, this band was preserved, although there was a slight variation, suggesting an interaction between buriti oil and LLDPE.

Comparing pure LLDPE samples with LLDPE/buriti compositions, a proximity in the degradation zones was observed, situated between 460 °C and 530 °C. This similarity indicates that the addition of buriti oil did not significantly compromise the thermal stability of the polymer. These results are consistent with previous studies on polymers and vegetable oils, where different degradation behaviors were attributed to specific characteristics of the materials [45]. The presence of functional groups in buriti oil, as evidenced in the FTIR analysis, may contribute to the observed thermal variation [33]. Comparative analysis with other polymers suggests that the additives may impact thermal stability [47,48,49]. Increases in Tonset and maximum temperatures (Tmax) may indicate an improvement in thermal stability, following patterns observed by other authors who used reinforcements in polymers [48].

In comparison with other biomass-based plasticizers, such as sorbitol used in PBAT/starch composites, buriti oil displayed slightly higher thermal stability. For instance, PBAT composites with sorbitol as a plasticizer exhibited multiple degradation steps, with a significant mass loss between 355 °C and 440 °C [50], which is lower than the Tonset of buriti oil. Furthermore, PLA composites plasticized with polyethylene glycol (PEG) showed a degradation profile where weight loss began at approximately 254.9 °C and full degradation occurred around 368 °C [51]. These degradation temperatures are also lower than those observed for LLDPE/buriti oil compositions. This suggests that buriti oil provides enhanced thermal stability compared to these other bio-based plasticizers, making it a more suitable option for applications requiring higher thermal resistance. Additionally, plasticizers based on cardanol in PVC demonstrated reduced thermal stability below 220 °C but improved stability above this temperature, due to the formation of char residue that shields the material during pyrolysis [52]. However, the decomposition temperature of buriti oil at 370–470 °C is notably higher, indicating better resistance to thermal degradation.

### 3.4. Differential Scanning Calorimetry (DSC)

**Figure 4:**
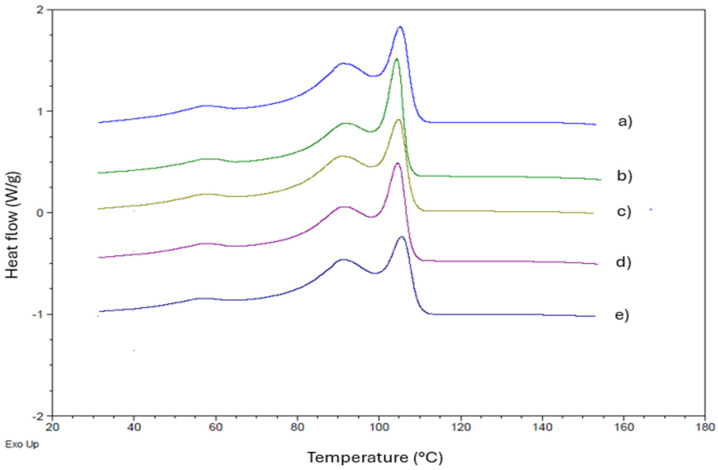
DSC analysis of LLDPE and its compositions with buriti oil: (**a**) LLDPE, (**b**) LLDPE/buriti oil—0.1%, (**c**) LLDPE/buriti oil—0.3%, (**d**) LLDPE/buriti oil—0.5%, (**e**) LLDPE/buriti oil—1.0%. Note: Crystallization temperature (Tc).

In the Tc curves depicted in Figure 4, we observe that the Tc1 values for the LLDPE and its compositions with buriti oil are approximately 105 °C, as shown in the corresponding Table 3. Tc represents the crystallization temperature, indicating the transition from the amorphous to the crystalline phase [53,54].

**Figure 5:**
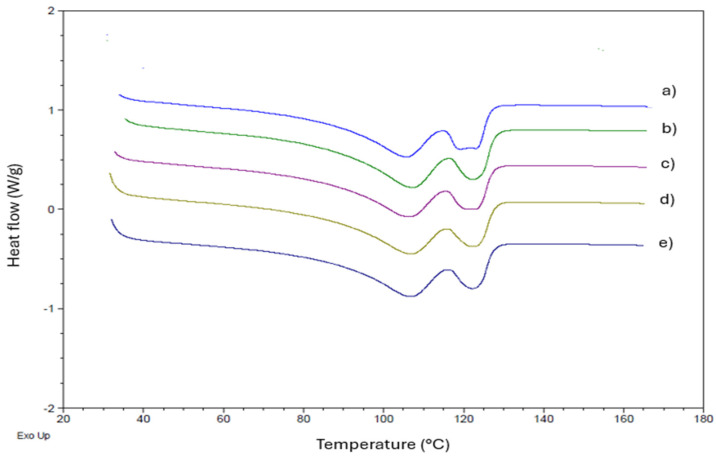
DSC analysis of LLDPE and its compositions with buriti oil: (**a**) LLDPE, (**b**) LLDPE/buriti oil—0.1%, (**c**) LLDPE/buriti oil—0.3%, (**d**) LLDPE/buriti oil—0.5%, (**e**) LLDPE/buriti oil—1.0%. Note: Crystalline melting temperature (Tm).

The DSC curves exhibit a bimodal endothermic peak for both the crystallization and melting processes, with no significant alterations in the values of crystalline melting temperatures (Tm). The crystallization thermograms display an intense exothermic peak, followed by a moderate peak and a weak signal for all compositions. This consistent behavior is observed across all samples, as depicted in Figure 5.

From the DSC results presented in Table 3, it is evident that there are no substantial changes in the crystallization temperatures (Tc) and melting temperatures (Tm) with the addition of low concentrations of buriti oil. This suggests the preservation of the analyzed thermal behavior. However, a slight decrease in the enthalpy of fusion (ΔHm) and the degree of crystallinity is observed in the compositions compared to pure LLDPE. This subtle reduction implies that the oil may be acting as a plasticizer, contributing as a processing aid. The DSC findings align with the torque rheometry processability curves, confirming the role of buriti oil as a processing aid.

The observed thermal properties, including crystallization and melting temperatures (Table 3), indicate the potential use of buriti oil as a processing aid without significant alteration in the overall thermal behavior of LLDPE. This behavior is consistent with previous studies investigating the plasticization of other polymers. For example, Murariu et al. [55] observed a more pronounced decrease in the glass transition temperature (Tg) and an increase in crystallinity in PLA compositions with bis(2-ethylhexyl) adipate (DOA), particularly at higher plasticizer concentrations (up to 20%). This differs from the results with buriti oil, where low concentrations did not induce substantial changes in thermal properties, possibly due to the saturation of the polymer matrix with small amounts of oil.

Our results are similar to some findings by Choi and Park [56], who, when studying PHBV with various plasticizers, reported that triethyl citrate (TEC) and dibutyl phthalate (DBP) were significantly more effective in reducing Tg and Tm than vegetable oils like epoxidized soybean oil (ESO). Although the plasticization effects on PHBV were more pronounced with plasticizers like TEC and DBP, buriti oil exhibits a pattern comparable to that observed with SO and ESO, where changes in thermal properties were less pronounced, indicating a milder interaction between the oil and the polymer matrix. These comparisons suggest that buriti oil may act similarly to less-effective vegetable oils as a plasticizer, offering processing advantages without causing significant changes in thermal characteristics.

### 3.5. Scanning Electron Microscopy (SEM)

**Figure 6:**
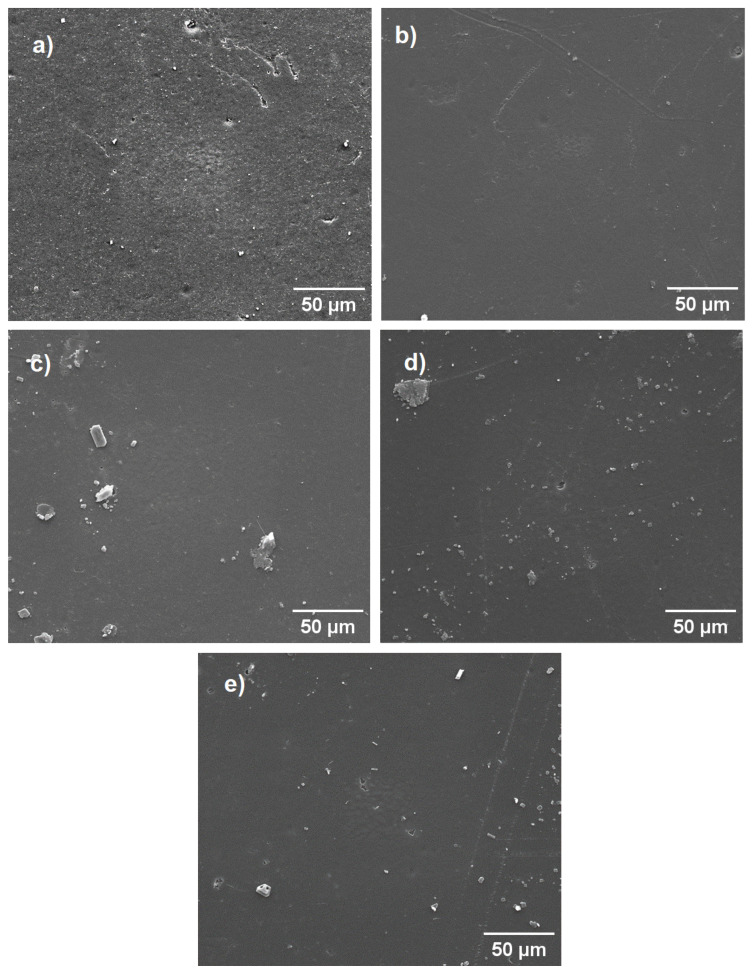
SEM images of LLDPE and LLDPE/buriti oil compositions: (**a**) Pure LLDPE showing rough surface morphology; (**b**) LLDPE/buriti oil composition displaying smoother and more homogeneous surface; (**c**–**e**) LLDPE/buriti oil compositions with dispersed particles of varying sizes, indicating partial agglomeration within the matrix.

The morphological characteristics of pure LLDPE and the LLDPE/buriti oil systems were analyzed by SEM, as presented in Figure 6. The micrograph of pure LLDPE is shown in Figure 6a. A surface characterized by high roughness was noted, a morphological characteristic of the polymer that is also highly influenced by the methodology used in the preparation of the specimens. The morphologies identified in Figure 6b refer to the systems containing buriti oil. These micrographs showed smoother and more-homogeneous surfaces regardless of the oil content added. Therefore, it was observed that the buriti oil used as a plasticizer during processing helped in the processing and contributed to obtaining smoother and more-homogeneous surfaces compared to the pure polymer.

The SEM images of the LLDPE/buriti oil compositions reveal several critical aspects of the material’s microstructure and dispersion characteristics. The micrographs exhibit a relatively uniform surface morphology, albeit with some irregularities and dispersed particles. In particular, the Figure 6a of the first set shows a smooth surface with minor surface roughness and scattered particles, while the Figure 6b similarly depicts a homogeneous surface interrupted by a few particles. Notably, all compositions exhibit the same pattern in the SEM analysis, indicating the consistent dispersion of buriti oil within the LLDPE matrix.

The second set of images presents a more detailed view of the particle dispersion within the LLDPE matrix. Both Figure 6c,d, indicate the presence of larger particles distributed throughout the polymer matrix. These particles vary in size and shape, suggesting that the mixing process may not have achieved perfect homogeneity. The heterogeneity in particle size and distribution could be indicative of incomplete mixing or the agglomeration of the buriti oil within the LLDPE matrix.

The overall surface morphology appears less brittle and more uniform in the LLDPE/buriti oil composites, which correlates with the improved mechanical properties observed in tensile testing. The presence of dispersed buriti oil particles likely contributes to the enhancement of mechanical properties, such as increased tensile strength and elongation at break. The uniformity in the microstructure, as evidenced by the SEM images, supports the notion that buriti oil acts as a plasticizer, improving the material’s flexibility and workability [57,58]. However, the presence of larger, heterogeneous particles suggests that further optimization of the mixing process is necessary to achieve a more uniform dispersion of buriti oil within the LLDPE matrix. This could involve adjustments to the mixing parameters or the introduction of additional processing steps to reduce particle size and improve distribution.

### 3.6. Contact Angle Analysis

The measurement of the contact angle serves as a crucial characterization tool, providing insights into the solid surface (substrate) properties. This technique involves determining the angle in degrees formed by a liquid droplet on the surface of the analyzed sample. The obtained angle value is directly related to the surface tension between the interfaces of the analyzed substances. Thus, the response indicates the substrate’s affinity for the analyzed liquid, representing its wettability [59,60]. In this study, water was used as the liquid, and the influence of increasing concentrations of buriti oil in the LLDPE matrix composition was assessed through contact angle measurements, as presented in Table 4. Although there are statistically significant differences in the results, the standard deviation values showed considerable amplitudes, indicating no alteration in the contact angle between pure LLDPE matrix and compositions containing up to 0.5% buriti oil by mass. However, the value obtained for the composition containing 1.0% oil by mass exhibited a slight increase in the angle, suggesting that buriti oil favored a reduction in wettability compared to other systems.

### 3.7. Mechanical Properties

**Figure 7:**
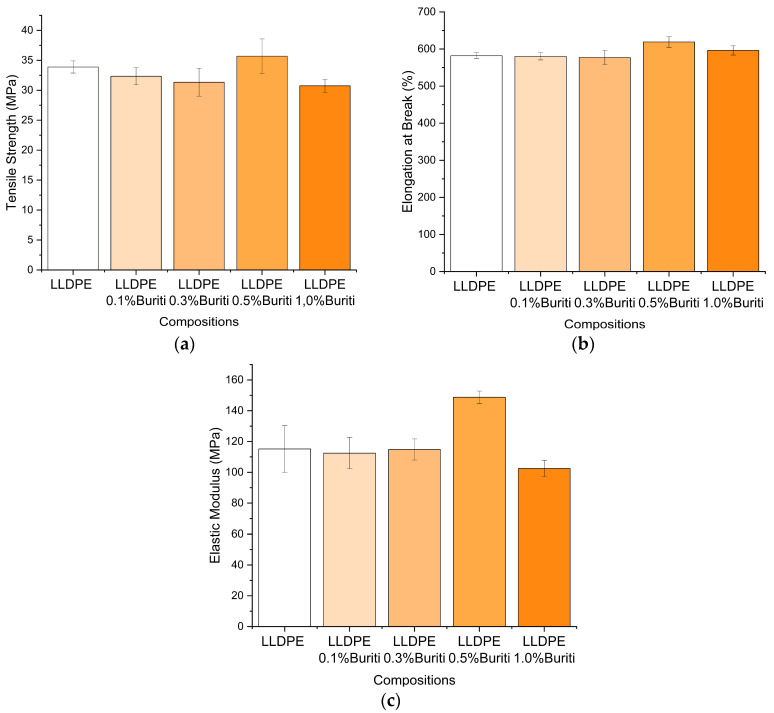
Effect of buriti oil addition on the mechanical properties of LLDPE composites, including tensile strength (**a**), elongation at break (**b**), and elastic modulus (**c**). Error bars represent standard deviations.

The mechanical properties, including the tensile modulus of elasticity, tensile strength, and elongation at break, were evaluated in accordance with ASTM D638 standards [61]. The results of the tensile test for LLDPE with increasing concentrations of buriti oil (0.1%, 0.3%, 0.5%, and 1%) are presented in Figure 7. The values represent the average of the obtained results with their respective standard deviations.

The tensile strength results (Figure 7) show that the addition of buriti oil influences the tensile behavior of LLDPE. The composition with 0.5% buriti oil exhibited the highest tensile strength, reaching approximately 35 MPa, which is a significant improvement compared to the other concentrations. This indicates that at this concentration, buriti oil enhances the strength of LLDPE, possibly due to better stress distribution and reduced chain mobility, leading to an overall stronger material. In a similar vein, Ngo et al. [62] discovered that introducing natural oils such as linseed or pine oil into natural-fiber-reinforced polyester composites predominantly diminishes surface microhardness and tensile properties but simultaneously augments ductility, indicating a complex interplay between natural oil additives and the mechanical properties of polymer composites.

The modulus of elasticity (Figure 7) follows a similar trend, where the 0.5%-buriti-oil composition shows a noticeable increase. The enhancement in modulus suggests that buriti oil at this concentration reinforces the polymer matrix, making it stiffer and more resistant to deformation under stress. This improvement in stiffness can be attributed to the interaction between the buriti oil and the LLDPE matrix, which may create a more rigid network structure.

Elongation at break (Figure 7) provides insights into the material’s flexibility and ductility. The results indicate that the incorporation of buriti oil generally enhances the elongation at break, with the 1%-buriti-oil composition showing the highest value. This suggests that buriti oil acts as a plasticizer, increasing the flexibility and ductility of the LLDPE matrix. The increased elongation at break signifies that the material can undergo more deformation before failure, contributing to improved toughness. Corroboratively, Brunel et al. [63] reported that natural additives, when incorporated into polymers, act as efficient plasticizers. In their study, concentrations of 5%, 7%, and 10% plasticizer were used, resulting in significant improvements in the mechanical properties of PHBV, including a reduction in the elastic modulus and increases in both impact resistance and elongation, highlighting the beneficial effects of these additives on the mechanical behavior of polymers. However, in the case of LLDPE with buriti oil, the improvements in mechanical properties were more subtle. Even with the addition of 0.5% buriti oil, the increases in the elastic modulus, tensile strength, and elongation at break were modest, suggesting that buriti oil, at low concentrations, acts more as a processing aid rather than significantly altering the mechanical properties.

Compared to other studies, the use of such a low concentration (1%) of buriti oil is noteworthy. For example, Râpă et al. [46] used concentrations of 10% to 30% of acetyl tributyl citrate (ATBC), along with DBEEA and TNC, in PLA, which resulted in increased elongation at break and decreased tensile strength and Young’s modulus, particularly at the higher concentrations of 20% and 30%. At the lower concentration, the variations were more modest. In the study by Choi and Park [56], PHBV plasticized with 20% triethyl citrate (TEC), soybean oil (SO), dibutyl phthalate (DBP), or epoxidized soybean oil (ESO) also showed significant increases in elongation, but with reductions in tensile strength and elastic modulus. Vegetable oils, such as soybean oil and epoxidized soybean oil, altered PHBV less compared to conventional plasticizers like DBP and TEC.

In contrast, the results with only 1% buriti oil showed that although the mechanical properties were not significantly altered, there was a substantial reduction in the torque required during processing, as discussed earlier, indicating improved processability. Previous studies did not explore plasticizer concentrations below 5%, which suggests that the use of low concentrations of buriti oil may present an advantage. Using less oil preserved the essential mechanical properties of LLDPE while also reducing the energy required for processing. This demonstrates the potential of buriti oil as a plasticizer in low concentrations, without the negative effects observed in other polymers with higher plasticizer concentrations.

## 4. Conclusions

This comprehensive analysis conducted on the LLDPE/buriti oil composites has provided valuable insights into their thermal, rheological, and mechanical behaviors. The FTIR spectrum of buriti oil reveals the presence of functional groups, such as C=O, which correspond to components like saturated and unsaturated fatty acids that are crucial for its plasticizing function. These results align with the existing literature, which characterizes buriti oil as being primarily composed of triglycerides and fatty acids, responsible for enhancing the flexibility and processability of LLDPE. By connecting these findings with studies on other vegetable oil plasticizers, we can assert that buriti oil follows a similar trend, functioning as an efficient bio-based plasticizer due to its physical interactions with the polymer matrix. The thermal analysis, including TGA and DTG, revealed distinct decomposition patterns, with the introduction of buriti oil influencing the onset temperatures and decomposition ranges. These findings align with the existing literature on the thermal behavior of similar polymer-oil composites, highlighting the significance of the observed trends. Examining the DSC results shed light on the crystallization and melting characteristics of the composites. The variations in crystallization and melting temperatures, as well as enthalpy changes, demonstrated the impact of buriti oil on the polymer’s molecular arrangement. This information is vital for understanding the material’s processing and potential applications. The torque-specific energy–time analysis offered valuable insights into the processing behavior of the composites. The reduction in torque values with the addition of buriti oil indicated a decrease in viscosity, while the observed peaks and subsequent decline suggested complex interactions during the mixing process. These aspects are crucial for optimizing the processing parameters for composite production. Mechanical property evaluation provided a comprehensive understanding of how buriti oil influences the tensile strength, elongation at break, and elastic modulus of LLDPE, with the 0.5% concentration showing the most promising results in terms of tensile strength and modulus of elasticity, while the 1% concentration excelled in elongation at break. The SEM analysis provided valuable insights into the microstructural characteristics of LLDPE/buriti oil composites. The findings indicate that while the buriti oil is generally well-dispersed and contributes positively to the mechanical properties, there is room for improvement in achieving a more homogeneous distribution. These microstructural observations align with the overall enhancement in processability and mechanical performance, reinforcing the potential of buriti oil as an effective bio-based additive in polymer composites. In summary, this systematic investigation of the LLDPE/buriti oil composites encompassed various aspects, offering a holistic perspective on their thermal, rheological, and mechanical characteristics. These findings contribute to the growing body of knowledge on bio-based polymer composites, providing valuable information for further optimization and potential applications in diverse industrial sectors.

## Figures and Tables

**Table 1:** Composition of LLDPE/buriti oil studied.

Samples	LLDPE	Buriti Oil (wt%)
LLDPE	100	-
LLDPE/BO-0.1	100	0.1
LLDPE/BO-0.3	100	0.3
LLDPE/BO-0.5	100	0.5
LLDPE/BO-1	100	1.0

**Table 2:** Torque (Nm) and specific energy (J/g) processability values.

Samples	T (Nm)	e (J/g)
LLDPE	11.4	838
LLDPE/BO-0.1	10.9	788.1
LLDPE/BO-0.3	10.8	791.8
LLDPE/BO-0.5	10.8	804.9
LLDPE/BO-1.0	10.7	768.4

**Table 3:** Detailed analysis of LLDPE composition with buriti oil, including effects on crystallization and thermal properties.

Samples	T_c1_ (°C)	T_c2_ (°C)	T_c3_ (°C)	T_m1_ (°C)	T_m2_ (°C)	ΔH_m_ (J/g)	X_c_ (%)
LLDPE	105.3	91.2	57.7	107.6	122.1	114.2	39.0
LLDPE/BO-0.1	104.4	91.7	57.1	105.1	121.9	103.8	35.4
LLDPE/BO-0.3	104.8	90.9	57.7	107.8	122.5	106.2	36.6
LLDPE/BO-0.5	104.6	91.2	57.4	106.7	123.4	104.1	35.5
LLDPE/BO-1.0	105.5	91.2	56.9	107.7	122.2	103.8	35.4

**Table 4:** Contact angle measurements of LLDPE and LLDPE/buriti Oil compositions.

Sample	Contact Angle [°]	Image
LLDPE	85.25 (2.01)	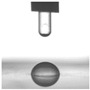
LLDPE/BO-0.1	87.08 (3.81)	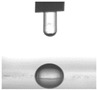
LLDPE/BO-0.3	88.74 (1.69)	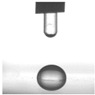
LLDPE/BO-0.5	89.06 (1.58)	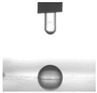
LLDPE/BO-1.0	100.96 (3.44)	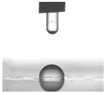

## Data Availability

The original contributions presented in the study are included in the article; further inquiries can be directed to the corresponding author.

## References

[1] Vieira M.G.A., da Silva M.A., dos Santos L.O., Beppu M.M. (2011). Natural-based plasticizers and biopolymer films: A review. Eur. Polym. J..

[2] Rahman M., Brazel C.S. (2004). The plasticizer market: An assessment of traditional plasticizers and research trends to meet new challenges. Prog. Polym. Sci..

[3] Marturano V., Marotta A., Salazar S.A., Ambrogi V., Cerruti P. (2023). Recent advances in bio-based functional additives for polymers. Prog. Mater. Sci..

[4] Oh E., Kim B.-h., Suhr J. (2022). A Trend and Market in Eco-friendly Plasticizers: Review and Prospective. Compos. Res..

[5] Byun Y., Zhang Y., Geng X., Han J.H. (2014). Chapter 5—Plasticization and Polymer Morphology. Innovations in Food Packaging.

[6] Xie C., Li H., Li L., Yu S., Liu F. (2008). Synthesis of plasticizer ester using acid-functionalized ionic liquid as catalyst. J. Hazard. Mater..

[7] Narvaéz Rincón P., Suárez Palacios O., Palsule S. (2015). Plasticizers. Polymers and Polymeric Composites: A Reference Series.

[8] The European Council for Plasticizers and Intermediates (2014). Plasticizers and flexible PVC.

[9] Gu J.-D., Wu E.K.W. (2021). Biodegradability of Synthetic Plastics and Polymeric Materials: An Illusion or Reality in Waste Management?. Appl. Environ. Biotechnol..

[10] Wei X.F., Linde E., Hedenqvist M.S. (2019). Plasticiser Loss from Plastic or Rubber Products through Diffusion and Evaporation. Npj Mater. Degrad..

[11] Jia P., Xia H., Tang K., Zhou Y. (2018). Plasticizers Derived from Biomass Resources: A Short Review. Polymers.

[12] Rahman M., Oßwald K., Langer B., Reincke K., Heinrich G., Kipscholl R., Stoček R. (2020). Influence of Plasticizers Basing on Renewable Sources on the Deformation and Fracture Behaviour of Elastomers. Fatigue Crack Growth in Rubber Materials.

[13] Durães J.A., Drummond A.L., Pimentel T.A.P.F., Murta M.M., Moreira S.G.C., Sales M.J.A. (2008). Thermal and structural behavior of Buriti oil/poly(methyl methacrylate) and Buriti oil/polystyrene materials. J. Therm. Anal. Calorim..

[14] de F. Silva M., Lopes P.S., da Silva C.F., Yoshida C.M.P. (2016). Active packaging material based on buriti oil—*Mauritia flexuosa* L. f. (*Arecaceae*) incorporated into chitosan films. J. Appl. Polym. Sci..

[15] Bispo A.G., Oliveira N.A., Cardoso C.X., Lima S.A.M., Job A.E., Osorio-Román I.O., Danna C.S., Pires A.M. (2018). Red-light- emitting polymer composite based on PVDF membranes and *Europium phosphor* using Buriti Oil as plasticizer. Mater. Chem. Phys..

[16] Sanches S., Silva-Júnior J., Ribeiro-Costa R. (2021). O uso dos óleos vegetais na prevenção do envelhecimento da pele. Res. Soc. Dev..

[17] Schlemmer D., Sales M.J.A., Resck I.S. (2010). Preparação, caracterização e degradação de blendas PS/TPS usando glicerol e óleo de buriti como plastificantes. Polímeros.

[18] Mohamed N., Othman N., Khimi R., Hayeemasae N. (2023). Perspective on opportunities of bio-based processing oil to rubber industry: A short review. Iran. Polym. J..

[19] Roy K., Poompiew N., Pongwisuthiruchte A., Potiyaraj P. (2021). Application of Different Vegetable Oils as Processing Aids in Industrial Rubber Composites: A Sustainable Approach. ACS Omega.

[20] Samarth N., Mahanwar P. (2015). Modified Vegetable Oil Based Additives as a Future Polymeric Material—Review. Open J. Org. Polym. Mater..

[21] Jarray A., Gerbaud V., Hemati M. (2016). Polymer-plasticizer compatibility during coating formulation: A multi-scale investigation. Prog. Org. Coat..

[22] Jarnthong M., Lopattananon N., Li Y., Yu H., Wang R., Wang Y., Liu H., Liao L., Peng Z. (2024). Performance of Moringa Oil as an Effective Bioplasticizer on Static and Dynamic Mechanical Properties of Natural Rubber Vulcanizates. ACS Sustain. Chem. Eng..

[23] Whelan T., Goff J. (1990). Plastics and Polymers. Injection Molding of Thermoplastics Materials—1.

[24] Borah J., Chaki T. (2011). Thermogravimetric and dynamic mechanical analysis of LLDPE/EMA blends. J. Therm. Anal. Calorim..

[25] Li B., He J. (2004). Investigation of mechanical property, flame retardancy and thermal degradation of LLDPE—Wood-fibre composites. Polym. Degrad. Stab..

[26] Yoon J.S., Lee D.H., Park E.S., Lee I.M., Park D.K., Jung S.O. (2000). Thermal and mechanical properties of ethylene/alpha-olefin copolymers produced over (2-MeInd)2ZrCl2/MAO system. Polymer.

[27] Mensah B., Onwona-Agyeman B., Nyankson E., Bensah D.Y. (2023). Effect of palm oil as plasticizer for compounding polar and non-polar rubber matrix reinforced carbon black composites. J. Polym. Res..

[28] Burns K., Ingram I.D.V., Potgieter J.H., Potgieter-Vermaak S. (2023). Synthesis and performance evaluation of novel soybean oil-based plasticisers for polyvinyl chloride (PVC). J. Appl. Polym. Sci..

[29] (2023). Standard Test Method for Melt Flow Rates of Thermoplastics by Extrusion Plastometer.

[30] Mizerovskii L.N., Afanas’eva V.V., Lytkina N.I. (1996). Melting of binary mixtures of low-density polyethylene and alkylbenzenes. Fibre Chem..

[31] Albuquerque M., Guedes I., Alcantara P., Moreira S. (2003). Infrared absorption spectra of Buriti (*Mauritia flexuosa* L.) oil. Vib. Spectrosc..

[32] Pereira de Oliveira J., Almeida O.P., Campelo P.H., Carneiro G., de Oliveira Ferreira Rocha L., Santos J.H.M., Gomes da Costa J.M. (2022). Tailoring the physicochemical properties of freeze-dried buriti oil microparticles by combining inulin and gum Arabic as encapsulation agents. LWT.

[33] Azevedo G.M.M. (2018). Nanoencapsulação de óleo de Buriti (*Mauritia flexuosa*) em Alginato e Gelatina: Caracterização e Avaliação da Solubilidade e Potencial Antimicrobiano. Master’s Thesis.

[34] Serpe G., Jarrin J., Dawans F. (1990). Morphology-processing relationships in polyethylene-polyamide blends. Polym. Eng. Sci..

[35] Wang C., Wang J., Yu C., Wu B., Wang Y., Li W. (2014). A novel method for the determination of steady-state torque of polymer melts by HAAKE MiniLab. Polym. Test..

[36] Marcelino G., Hiane P.A., Pott A., de Oliveira Filiú W.F., Caires A.R.L., Michels F.S., Júnior M.R.M., Santos N.M.S., Nunes A.A., Oliveira L.C.S. (2022). Characterization of Buriti (*Mauritia flexuosa*) Pulp Oil and the Effect of Its Supplementation in an In Vivo Experimental Model. Nutrients.

[37] Silva S.M., Sampaio K.A., Taham T., Rocco S.A., Ceriani R., Meirelles A.J.A. (2009). Characterization of Oil Extracted from Buriti Fruit (*Mauritia flexuosa*) Grown in the Brazilian Amazon Region. J. Am. Oil Chem. Soc..

[38] Khalaf A.I., Ward A.A., Abd El-Kader A.E., El-Sabbagh S.H. (2015). Effect of selected vegetable oils on the properties of acrylonitrile-butadiene rubber vulcanizates. Polimery.

[39] O’Brien R.D. (2009). Fats and Oils: Formulating and Processing for Applications.

[40] Hosney H., Nadiem B., Ashour I., Mustafa I., El-Shibiny A. (2018). Epoxidized vegetable oil and bio-based materials as PVC plasticizer. J. Appl. Polym. Sci..

[41] Hassan A.A., Abbas A., Rasheed T., Bilal M., Iqbal H.M.N., Wang S. (2019). Development, influencing parameters and interactions of bioplasticizers: An environmentally friendlier alternative to petro industry-based sources. Sci. Total Environ..

[42] Zhang H., Zhu F., Fu Q., Zhang X., Zhu X. (2019). Mechanical properties of renewable plasticizer based on ricinoleic acid for PVC. Polym. Test..

[43] Wang M., Song X., Jiang J., Xia J., Ding H., Li M. (2018). Plasticization and thermal behavior of hydroxyl and nitrogen rich group-containing tung-oil-based ester plasticizers for PVC. New J. Chem..

[44] Zhang Z., Jiang P., Liu D., Feng S., Zhang P., Wang Y., Fu J., Agus H. (2021). Research progress of novel bio-based plasticizers and their applications in poly(vinyl chloride). J. Mater. Sci..

[45] Escalante J., Chen W.H., Tabatabaei M., Hoang A.T., Kwon E.E., Andrew Lin K.Y., Saravanakumar A. (2022). Pyrolysis of lignocellulosic, algal, plastic, and other biomass wastes for biofuel production and circular bioeconomy: A review of thermogravimetric analysis (TGA) approach. Renew. Sustain. Energy Rev..

[46] Rapa M., Nita R.N.D., Vasile C. (2017). Influence of Plasticizers Over Some Physico-chemical Properties of PLA. Mater. Plast..

[47] Gong J., Niu R., Liu J., Chen X., Wen X., Mijowska E., Sun Z., Tang T. (2014). Simultaneously improving the thermal stability, flame retardancy and mechanical properties of polyethylene by the combination of graphene with carbon black. RSC Adv..

[48] Ríos-Soberanis C.R., Collí-Pacheco J.P., Estrada-León R.J., Moo-Huchin V.M., Yee-Madeira H.T., Pérez-Pacheco E. (2021). Biocomposites based on plasticized starch: Thermal, mechanical and morphological characterization. Polym. Bull..

[49] Hernández Berrío Y.D.C., Realpe Jiménez Á., De Ávila Montiel G. (2022). Effect of glycerol, sunflower oil, and glucose on the physico-chemical and mechanical properties of chitosan/polyvinyl alcohol-based films. Polym. Bull..

[50] Perumal N., Sreekantan S., Hamid Z.A.A. (2024). Effect of Plasticizer and Compatibilizer on Properties of Polybutylene Adipate-Co-Terephthalate (PBAT) with Acetylated Starch. J. Polym. Environ..

[51] Norazlina H., Kamal Y. (2021). Elucidating the plasticizing effect on mechanical and thermal properties of poly(lactic acid)/carbon nanotubes nanocomposites. Polym. Bull..

[52] Jia P., Zhang M., Hu L., Wang R., Sun C., Zhou Y. (2017). Cardanol Groups Grafted on Poly(vinyl chloride)—Synthesis, Performance and Plasticization Mechanism. Polymers.

[53] Li D., Zhou L., Wang X., He L., Yang X. (2019). Effect of Crystallinity of Polyethylene with Different Densities on Breakdown Strength and Conductance Property. Materials.

[54] Dubdub I., Al-Yaari M. (2020). Pyrolysis of Low Density Polyethylene: Kinetic Study Using TGA Data and ANN Prediction. Polymers.

[55] Murariu M., Da Silva Ferreira A., Alexandre M., Dubois P. (2008). Polylactide (PLA) designed with desired end-use properties: 1. PLA compositions with low molecular weight ester-like plasticizers and related performances. Polym. Adv. Technol..

[56] Choi J., Park W.H. (2003). Effect of biodegradable plasticizers on thermal and mechanical properties of poly(3-hydroxybutyrate). Polym. Test..

[57] Liang Z., Tan Z., Hong R., Ouyang W., Yuan J., Zhang C. (2023). Automatically Predicting Material Properties with Microscopic Images: Polymer Miscibility as an Example. J. Chem. Inf. Model..

[58] Ali A., Zhang N., Santos R.M. (2023). Mineral Characterization Using Scanning Electron Microscopy (SEM): A Review of the Fundamentals, Advancements, and Research Directions. Appl. Sci..

[59] Atmakuri A., Janušas G., Siddabathula M., Palevicius A. Wettability and Moisture Analysis on Natural Fiber Reinforced Epoxy Resin Hybrid Composites. Proceedings of the 2020 International Conference Mechatronic Systems and Materials (MSM).

[60] Agrawal G., Negi Y., Pradhan S., Dash M., Samal S., Tanzi M.C., Farè S. (2017). 3–Wettability and contact angle of polymeric biomaterials. Characterization of Polymeric Biomaterials.

[61] (2022). Standard Test Method for Tensile Properties of Plastics.

[62] Ngo T.T., Lambert C.A., Bliznyuk M., Kohl J.G. (2013). Effect of a Tertiary Oil Phase on the Mechanical Properties of Natural Fiber-Reinforced Polyester Composites. Polym.-Plast. Technol. Eng..

[63] Brunel D.G., Pachekoski W.M., Dalmolin C., Agnelli J.A.M. (2014). Natural additives for poly (hydroxybutyrate-CO-hydroxyvalerate)-PHBV: Effect on mechanical properties and biodegradation. Mater. Res..

